# Acid-Base and Electrolyte Status during Normovolemic Hemodilution with Succinylated Gelatin or HES-Containing Volume Replacement Solutions in Rats

**DOI:** 10.1371/journal.pone.0072848

**Published:** 2013-09-02

**Authors:** Johanna K. Teloh, Katja B. Ferenz, Frank Petrat, Christian Mayer, Herbert de Groot

**Affiliations:** 1 University of Duisburg-Essen, Institute of Physiological Chemistry, University Hospital Essen, Essen, Germany; 2 University of Duisburg-Essen, Institute of Physical Chemistry, Essen, Germany; National Institutes of Health, United States of America

## Abstract

**Background:**

In the past, several studies have compared different colloidal replacement solutions, whereby the focus was usually on the respective colloid. We therefore systematically studied the influence of the carrier solution’s composition of five approved colloidal volume replacement solutions (Gelafundin, Gelafusal, Geloplasma, Voluven and Volulyte) on acid-base as well as electrolyte status during and following acute severe normovolemic hemodilution. The solutions differed in the colloid used (succinylated gelatin vs. HES) and in the presence and concentration of metabolizable anions as well as in their electrolyte composition.

**Methods:**

Anesthetized Wistar rats were subjected to a stepwise normovolemic hemodilution with one of the solutions until a final hematocrit of 10%. Subsequent to dilution (162 min), animals were observed for an additional period (150 min). During dilution and observation time blood gas analyses were performed eight times in total. Additionally, in the Voluven and Volulyte groups as well as in 6 Gelafundin animals, electrolyte concentrations, glucose, pH and succinylated gelatin were measured in urine and histopathological evaluation of the kidney was performed.

**Results:**

All animals survived without any indications of injury. Although the employed solutions differed in their respective composition, comparable results in all plasma acid-base and electrolyte parameters studied were obtained. Plasma pH increased from approximately 7.28 to 7.39, the plasma K^+^ concentration decreased from circa 5.20 mM to 4.80-3.90 mM and the plasma Cl^−^ concentration rose from approximately 105 mM to 111–120 mM. Urinary analysis revealed increased excretion of K^+^, H^+^ and Cl^−^.

**Conclusions:**

The present data suggest that the carrier solution’s composition with regard to metabolizable anions as well as K^+^, Ca^2+^ only has a minor impact on acid-base and electrolyte status after application of succinylated gelatin or HES-containing colloidal volume replacement solutions.

## Introduction

Colloids are often added to solutions that are applied for volume replacement to establish or maintain a colloid osmotic pressure in the intravascular compartment after infusion [1]. Colloidal solutions, as an alternative to crystalloid solutions, have been recommended in several resuscitation guidelines [2], although still controversially discussed [3], [4]. Based on the affiliation of the colloid osmotic substances, a classification in natural (for example albumin) and synthetic colloids (i.e., dextran, hydroxyethyl starch (HES) or succinylated gelatin) can be made [1].

**Figure 1:**
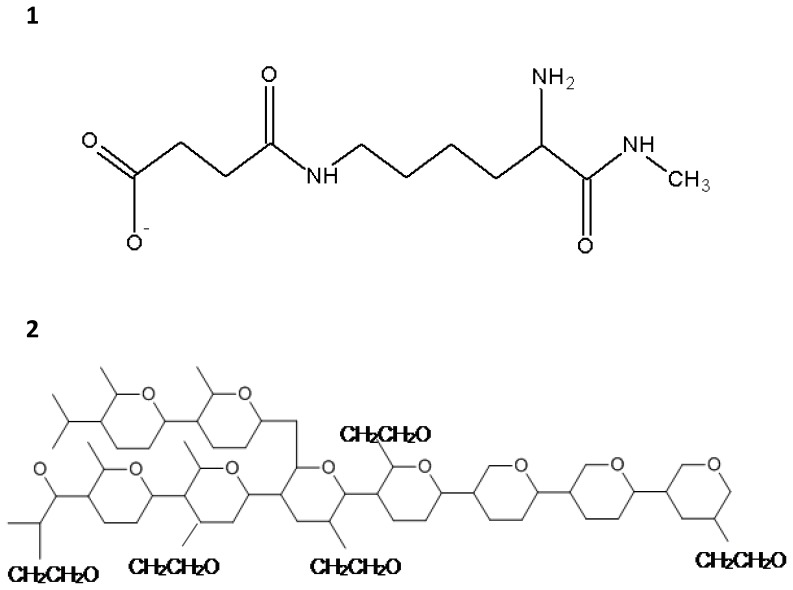
Chemical structure of the colloidal substances employed. 1) Succinylated gelatin chain whose terminal carboxyl group ist deprotonated at physiological pH. 2) Hydroxyethyl starch whose single glucose subunits are additionally hydroxyethylated at carbon atoms two, three and six.

For production of succinylated gelatin (modified fluid gelatin), bovine collagen is thermically degraded before succinic anhydride is added, which reacts during the process of succinylation with the gelatin’s basic amino groups [4], [5]. Hence, those groups are finally substituted with carboxyl groups being deprotonated at physiological pH (Figure 1). The introduction of the negatively charged succinyl groups, which are repelled by the similarly charged endothelial glycocalyx, plus the conformational changes in the succinylated gelatin’s molecular structure, result in an elevated intravascular dwell-time of solutions containing succinylated gelatin in comparison to unmodified gelatin.

HES is a highly branched starch polymer for which amylopectin serves as raw material [6]. The amylopectin’s basic glucose units are linked via α-1,4 glycosidic bonds [7]. Additional ramifications are realized via α-1,6 glycosidic bonds. For application as a component in a medical product, the native starch polymer first has to be degraded into smaller fragments, either enzymatically or via acid hydrolysis. Due to several reasons (for example gelatinization and rapid degradation by endogenous α-amylase), natural, unmodified starch cannot be employed as colloid osmotic active substance in a volume replacement solution [8]. Thus, the molecule experiences some modifications in order to enhance its properties *in vivo*. For this purpose, the starch molecule is hydroxyethylated at carbon atoms at the positions two, three and six within every glucose subunit, using amylopectin and ethylene oxide in an alkaline setting [9]. That way, water solubility is increased [8] and cleaveage by α-amylase is delayed, but not completely prevented, resulting in a prolonged volume effect [7], [9]. Nonetheless, HES molecules are enzymatically degraded until the fragments are small enough to pass the renal filtration barrier and are finally excreted. In contrast to succinylated gelatin, the starch polymer is neither deprotonated nor protonated at physiological pH, thus being uncharged (Figure 1).

Colloid osmotic substances are dissolved in an aqueous solution, the so-called carrier solution. To ensure an unproblematic substitution, the current carrier solutions are approximately isoosmotic. They contain, however, the various electrolytes (Na^+^, K^+^, Ca^2+^ and Cl^−^) in different concentrations. Furthermore, some carrier solutions additionally include organic anions such as lactate and acetate. These so-called metabolizable anions are able to counteract acidosis due to the unavoidable dilution of bicarbonate upon application of the bicarbonate-free volume substitutes. Per anion metabolized, one H^+^ is consumed and one HCO_3_^−^ is generated. Thus, the physiological pH is considered to be especially preserved by those solutions containing a metabolizable anion [10] as long as sufficient oxygen is provided [11]. Solutions mimicking physiological concentrations of various ions in plasma, including metabolizable anions for bicarbonate substitution, are called balanced solutions, whereas pure saline-based carrier solutions containing rather unphysiological concentrations of Na^+^ and Cl^−^ are named unbalanced.

In the past, several studies have been performed of which many exhibited either a clinical background or were *in vitro* studies. This implicates that hemodilutions were rather moderate in the clinical setting [12], [13] or display the disadvantages of an *in vitro* situation [14], [15] and are therefore not directly applicable. Those in total heterogeneous studies compared either crystalloid with colloidal volume replacement solutions [16], [17] or several colloidal replacement solutions containing different colloids in different clinical settings (for example HES vs. gelatin [12], [18], dextran vs. HES vs. albumin [13], albumin vs. gelatin vs. HES [19]) as regards mortality or the requirement of kidney transplantation but never the focus was solely on acid-base status. A few also analysed the influence of balanced or unbalanced carrier solutions but only with HES as colloid osmotic substance [20]–[23]. The carrier solution’s impact in the presence of succinylated gelatin as identical colloid has never been analyzed to our knowledge.

To study the carrier solution’s impact in the presence of succinylated gelatin as osmotically active substance on acid-base and electrolyte status, we employed three already approved solutions for volume replacement, i.e., Gelafundin, Gelafusal and Geloplasma, differing by the presence and concentration of electrolytes and metabolizable anions (Table 1) in a model of severe normovolemic hemodilution to a final hematocrit of 10%. To examine the different influence of the colloid osmotic substance itself, we also included two approved HES solutions (i.e., Voluven and Volulyte). The model of severe normovolemic hemodilution was chosen to substitute a maximal amount of fluid and thus to enforce a marked change in the parameters studied without any influence of pathological mechanisms like centralizing of circulation or stop of aerobic glycolysis both present in hypovolemia and hemorrhagic shock.

**Table 1:** Composition of the five volume replacement solutions Gelafundin, Gelafusal, Geloplasma, Voluven and Volulyte.

	Gelafundin	Gelafusal	Geloplasma	Voluven	Volulyte
colloid osmoticactive substance	Succinylatedgelatin	Succinylatedgelatin	Succinylatedgelatin	Hydroxyethylstarch	Hydroxyethylstarch
**quantity [g/L]**	40	40	30	60	60
**real osmolality** **[mOsm/kg]**	265	270	273	304	283
	**molarity [mM]**				
**Na^+^**	154	130	150	154	137
**K^+^**	–	5.4	5	–	4.0
**Ca^2+^**	–	0.9	–	–	–
**Mg^2+^**	–	1.0	1.5	–	1.5
**Cl^−^**	120	85	100	154	110
**lactate**	–	–	30	–	–
**acetate**	–	27	–	–	34

Note: The apparent anion gap in the case of Gelafundin, Gelafusal and Geloplasma is compensated by the negative charge of succinylated gelatin.

## Methods

### Ethics Statement

Experiments were conducted in accordance with the standards of Annex III of the directive 2010/63/EU of the European Parliament and of the Council of 22 September 2010 on the protection of animals used for scientific purposes [24]. The experimental protocol was reviewed and approved by the local Animal Care and Use Committee (Animal Care Center, University of Duisburg-Essen, Essen, Germany, and the district government of Duesseldorf (“North Rhine-Westphalia State Environment Agency”, Recklinghausen, Germany), Germany) with a Permit Number 84-02.04.2012.A327, G1314/12) All surgery was performed under isoflurane anesthesia, and all efforts were made to minimize suffering.

### Animals

A total number of 36 male Wistar rats (*Rattus norvegicus*, 410 g –470 g) were obtained from the central animal unit of the Essen University Hospital. Animals were kept under standardized conditions of temperature (22°C ±1°C), humidity (55% ±5%), and 12- h/12- h light dark cycles. They were fed *ad libitum* (Ssniff-Spezialdiaeten, Soest, Germany) with free access to water and not fasted before the experiments.

### Chemicals/Materials

Gelafundin and NaCl solution (0.9%) was provided by B. Braun (Melsungen, Germany), Gelafusal by Serumwerk Bernburg (Bernburg, Germany) and Geloplasma, Voluven and Volulyte by Fresenius Kabi (Bad Homburg, Germany). Isoflurane (Forene) was obtained from Abbott (Wiesbaden, Germany), ketamine 10% from Ceva (Duesseldorf, Germany) and lidocaine (Xylocain 1%) from AstraZeneca (Wedel, Germany). Portex catheters (0.58 mm inner diameter, 0.96 mm outer diameter) were from Smiths Medical International (Hythe, UK). As urinary catheter an indwelling venous cannula was used (Vasofix, B. Braun, Melsungen, Germany). For dilution of urine, a special diluent was employed (Radiometer, Copenhagen, Denmark). Medical oxygen was from Air Liquide (Duesseldorf, Germany).

### Anesthesia, Analgesia, and Surgical Procedures

Rats were anesthetized with isoflurane (2.0% in 100% medical O_2_ at 4.0 l/min for induction, 1.0%–2.0% isoflurane in 100% medical O_2_ at 1.0 l/min throughout the experiment) through face masks connected to a vaporizer (Isofluran Vet. Med. Vapor; Draeger, Luebeck, Germany) and received ketamine (50 mg/kg body weight subcutaneously) into the right chest wall for analgesia. After local lidocaine administration (5 mg/kg body weight subcutaneously), a skin-deep inguinal incision of about 2 cm was made, and a Portex catheter (0.58 mm inner diameter, 0.96 mm outer diameter) was placed within the right femoral artery and the right femoral vein. For insertion of the urinary catheter, a median inguinal incision of about 2 cm was made with subsequent preparation of the urinary bladder. For the period of infusion of the volume replacement solution, the prolonged infusion of 0.9% NaCl-solution (5 ml/kg body weight x h, 37°C) to compensate intraoperative fluid depletion over surgical areas and the respiratory epithelium was interrupted. At the end of the experiment, the left kidney from animals of the Voluven and Volulyte group as well as from 6 animals of the Gelafundin group (see experimental groups) was harvested. All animals were sacrificed by resection of the heart under deep isoflurane anesthesia.

### Hemodilution

The model of normovolemic hemodilution was established according to Johannes and Young [25], [26], with some modifications.

Briefly, 3 ml blood was repetitively withdrawn until a final hematocrit of 10% ±1% was attained. Every withdrawal was followed by a 15- min pause interval for circulatory stabilization as established previously [25], [26]. The blood was taken from the arterial catheter at an approximate rate of 1 ml/min by hand [26]. The removed volume was simultaneously replaced over the venous catheter (1 ml/min) by one of the five volume replacement solutions using a syringe pump (Medfusion Inc, Raleigh, United States). After finishing hemodilution, animals were monitored for 150 min. If mean arterial blood pressure (MAP) had fallen below 60 mmHg, a bolus of 0.5 ml of the respective volume replacement solutions was given.

Hematocrit was measured with the help of a centrifuge (10 min, 22024.6 x g, 4°C; Universal 320R, Hettich, Tuttlingen, Germany). For this purpose, blood samples (0.3 ml) were taken from the femoral artery immediately before starting the next dilutional step using a 2- ml syringe (Pico50, Radiometer Medical ApS, Brønshøj, Denmark) containing 80 IU electrolyte-balanced heparin.

### Experimental Groups

One group (n = 6) received Gelafusal for volume replacement during hemodilution, the second group (n = 6) Geloplasma, the third (n = 6) Voluven, the fourth (n = 6) Volulyte and the fifth (n = 12) Gelafundin. Parameters of the five groups were compared.

All animals of the Voluven and Volulyte group as well as 6 animals of the Gelafundin group experienced the insertion of a urinary catheter for continous urine collection and further analysis (pH, electrolytes as well as succinate analysis).

### Biomonitoring

For close documentation of the established model, we monitored several systemic and vital parameters throughout the experiment. Systolic blood pressure, diastolic blood pressure and MAP were measured continuously by using the femoral artery catheter, which was connected to a pressure transducer, and displayed on a monitor. Ringer solution was infused at a rate of 3 ml/h to keep the catheter functional. Heart rates were determined from systolic blood pressure spikes. The breathing rate was determined according to the number of ventilatory movements in 15 seconds. The core body temperature of all rats was monitored using a rectal sensor and maintained around 37.3°C during the whole experiment by means of an underlying thermostat-controlled operating table and by covering the animal additionally with aluminum foil.

### Assessment of Blood Parameters

Using a 2- ml syringe containing 80 IU electrolyte-balanced heparin, blood samples (2 ml) were taken from the femoral artery (as part of the blood withdrawn for hemodilution) at the first, fourth, seventh and ninth dilutional step as well as 0.3 ml each at 15, 45, 90 and 150 min after dilution had been completed. Arterial oxygen and carbon dioxide partial pressure (pO_2_, pCO_2_), pH, base excess, electrolytes (Na^+^, K^+^, Ca^2+^ and Cl^−^) and metabolic parameters (lactate, glucose) were assessed with a blood gas analyzer (ABL 715, Radiometer, Copenhagen, Denmark).

### Assessment of Urinary Parameters

After insertion of the urinary catheter, the initial urine was removed and stored at −80°C until further examination. Both during the phase of dilution and during the observation time, urine was continuosly collected and again stored at −80°C. Na^+^, K^+^, Ca^2+^, Cl^−^ and glucose were analyzed with the help of a blood gas analyzer (ABL 715, Radiometer, Copenhagen, Denmark). Prior to these measurements, urine samples were diluted 1∶2 with urine diluent. To verify results obtained by the blood gas analyzer as regards the glucose amount in urine, we employed urine testing strips, confirming this data. The pH was measured with the help of an electrode.

All urine samples were submitted to quantitative analysis of succinylated gelatin by nuclear magnetic resonance (NMR). Without further sample preparation, ^1^H line spectra were measured using 5 mm sample tubes in a DRX 500 spectrometer (Bruker, Karlsruhe, Germany) by accumulation of 320 scans. An external reference of D_2_O (with traces of HDO) in a coaxial tube was used to create the lock signal. The free induction decay induced by a single 90 degree pulse for protons was accumulated and subsequently Fourier-transformed to generate a corresponding line spectrum. A waiting period of 7 seconds between the scans allowed for sufficient spin-lattice relaxation. The spectra were referenced to the proton signal of HDO to 4.8 ppm.

Using a corresponding spectrum of a commercial succinylated gelatin-containing solution (4%, Gelafundin), the signals at 2.394 ppm and at 2.433 ppm could be identified as deriving from the two different CH_2_-segments of the succinate residue connected to the gelatin chain. Both together, they served as markers for the presence of succinylated gelatin. The assignment of these peaks has been confirmed by ^1^H-^13^C-correlation spectroscopy and by comparison with the proton spectrum of sodium succinate solution. In the urine samples, only a single peak near 2.39 ppm could be detected, indicating that only free succinate (with two equivalent CH_2_-groups) was present. For a quantitative analysis, this section of the proton spectra was integrated and referenced to the combined signal intensities (I_G_ = I_2.394 ppm_+I_2.433 ppm_) of Gelafundin with a succinylated gelatin concentration of c_G_ = 4%. The succinate concentration for a given sample x was then calculated as c_x_ = c_G_ (I_x_/I_G_). Considering the possible presence of weak background signals and baseline distortions, an experimental uncertainty of ±10% was accepted.

Both the initial urine of the Gelafundin group as well as the one of Voluven group collected during observation time were estimated on the basis of 3 animals. Other mean values were calculated on the basis of 6 animals.

### Histopathologic Evaluation of the Kidney

The entire left kidney was fixed for 24 h to 48 h in formalin (10% neutral buffered). Paraffin-embedded sections (3 µm) were made and subsequently stained with hematoxylin-eosin and evaluated.

### Statistical Analysis

Experiments were performed with 6 animals per group (Gelafundin group: 12 animals). The data are expressed as mean values ± standard error of the mean (SEM). Comparisons among different time points within one group or among multiple groups at one time point were performed using one-way independent analysis of variance (ANOVA), whereas comparisons for an analysis over time were performed using repeated measurements ANOVA, both followed by the Fisher (LSD) post hoc analysis. A *P* value <0.05 was considered significant.

## Results

### Hematocrit, Blood Pressure and other Vital Parameters

**Figure 2:**
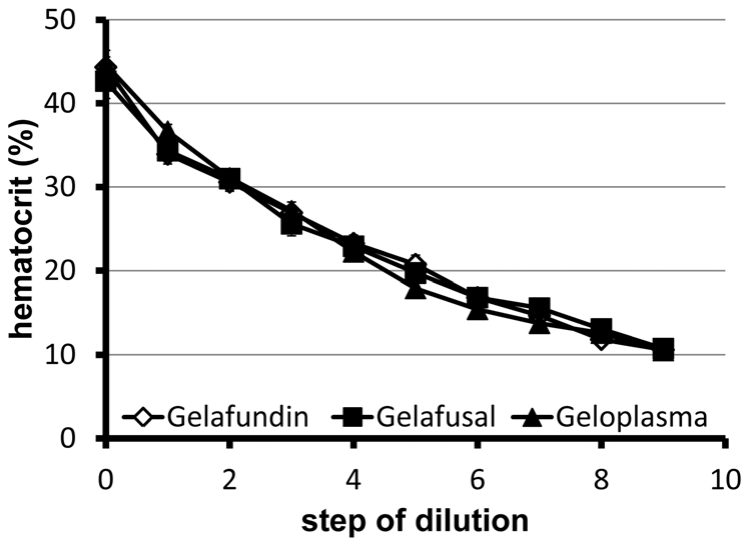
Effect of hemodilution on the current hematocrit. Rats underwent uniform normovolemic hemodilution to a final hematocrit of 10% with either Gelafundin, Gelafusal or Geloplasma by withdrawal of 3 ml blood per step. An interval of 15 min was left between the single steps. Hematocrit was determined in arterial blood samples immediately before the next withdrawal.

**Figure 3:**
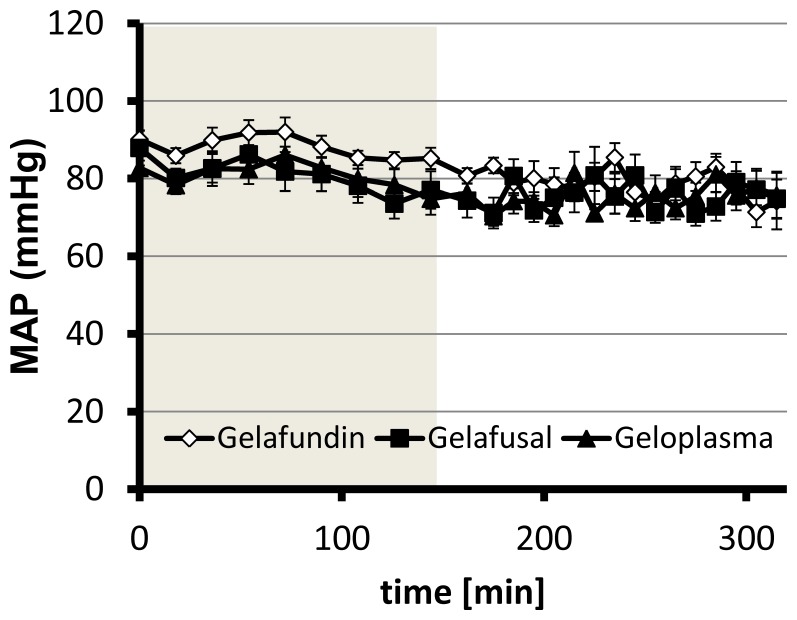
Influence of different volume replacement solutions on blood pressure during and subsequent to normovolemic hemodilution. Rats underwent normovolemic hemodilution to a final hematocrit of 10% for 162 min (phase of hemodilution: light grey) with either Gelafundin, Gelafusal or Geloplasma and were subsequently observed for 150 min. For analysis mean arterial blood pressure was monitored.

In the course of hemodilution, the hematocrit dropped steadily in the animals substituted with colloidal replacement solutions containing either succinylated gelatin (Figure 2) or solutions on HES basis (data not shown). Between the single groups no differences were detectable. The final hematocrit of 10% ±1% was reached after nine steps of dilution (162 min) in all groups. All animals survived the whole experimental time of hemodilution and the subsequent observation time (150 min). The MAP of all animals having received succinylated gelatin slightly decreased during the experiment, both during the phase of dilution and the observation time, but never fell below 70 mmHg (Figure 3). The same was true for the Voluven and Volulyte groups (data not shown). The heart and breathing rate remained constant over the experiment in all groups studied (data not shown).

### Acid-base and Metabolic Status

**Figure 4:**
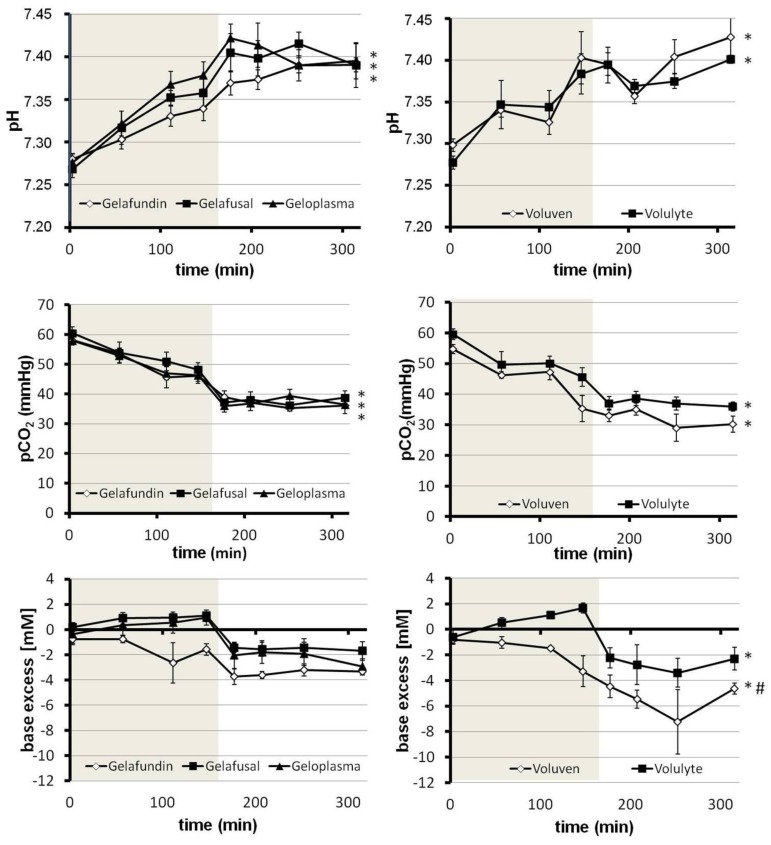
Impact of different volume replacement solutions on acid-base parameters during and subsequent to normovolemic hemodilution. Rats underwent normovolemic hemodilution to a final hematocrit of 10% for 162 min (phase of hemodilution: light grey) with either Gelafundin, Gelafusal, Geloplasma, Voluven or Volulyte and were subsequently observed for 150 min. Parameters were determined in arterial blood samples at the points indicated. * <0.05 compared with the value measured at 3 min, in the respective group. # <0.05 (Voluven vs. Volulyte).

In all groups, the plasma pH rose throughout the experiment (Figure 4), and there was no significant difference between the groups. The pH increased from values around 7.28 to 7.34 (Gelafundin), 7.38 (Volulyte), 7.40 (Voluven), 7.41 (Gelafusal) and 7.42 (Geloplasma), respectively, shortly after hemodilution had been completed. During the subsequent observation time, the pH of these groups stayed approximately constant to end up at a final value of 7.39 (Gelafundin and Gelafusal), 7.40 (Geloplasma and Volulyte) and 7.43 (Voluven), respectively.

Again in all groups, pCO_2_ decreased from initial values of 55 to 60 mmHg to values of 35 to 45 mmHg (Figure 4). During the observation time it dropped even further to values of 30 to 37 mmHg which were kept until the end of the experiment.

Throughout the experiment the pO_2_ remained almost constant around 400 mmHg in all animals (data not shown).

Starting from values around-1 to 0 mM, base excess slightly increased during hemodilution in the Gelafusal, Geloplasma and Volulyte groups or stayed constant (Gelafundin), but decreased a little in the Voluven group to-3 mM (Figure 4). Upon cessation of dilution, base excess dropped to-2 mM (Gelafusal, Geloplasma, Volulyte),-3 mM (Gelafundin) and-5 mM (Voluven), respectively, to stay at the respective level during the whole observation time.

The blood lactate concentration remained at levels below 1.9 mM in the Gelafundin, Gelafusal, Voluven and Volulyte groups, both during the phase of dilution and during the observation time, with the tendency to decrease towards the end of the experiment (Table 2). In contrast, over the same period of time, the lactate concentration in the Geloplasma group increased to 2.7 mM. The blood glucose concentration of all groups remained in the range between 175 to 225 mg/dl during the phase of dilution. During the observation time, the glucose concentration steadily decreased to end up at values of 146 mg/dl (Voluven), 133 mg/dl (Volulyte), 131 mg/dl (Gelafundin), 115 mg/dl (Gelafusal) and 112 mg/dl (Geloplasma), respectively.

**Table 2:** Influence of different volume replacement solutions (Gelafundin, Gelafusal, Geloplasma, Voluven, Volulyte) on lactate and glucose in arterial blood samples during and subsequent to normovolemic hemodilution in rats.

	Gelafundin		Gelafusal		Geloplasma		Voluven		Volulyte	
time(min)	lactate[mM]	glucose[mg/dl]	lactate[mM]	glucose[mg/dl]	lactate[mM]	glucose[mg/dl]	lactate[mM]	glucose[mg/dl]	lactate[mM]	glucose[mg/dl]
**3**	1.4±0.1	193.7±3.6	1.4±0.1	195.2±9.3	1.8±0.1	212. 0±14.3	1.3±0.1	218.5±7.4	1.3±0.1	218.8±12.4
**57**	1.4±0.1	190.8±3.5	1.3±0.1	186.3±6.1	1.62±0.1	199.2±9.4	1.6±0.4	205.7±10.7	1.3±0.1	213.0±19.3
**111**	1.4±0.1	193.3±8.0	1.4±0.1	183.7±7.6	1.9±0.1	198.8±12.6	1.5±0.2	223.7±11.5	1.3±0.2	216.3±11.8
**147**	1.4±0.1	191.8±9.8	1.6±0.1	179.8±5.1	2.2±0.2	212.7±16.1	1.7±0.2	204.5±11.2	1.6±0.2	217.7±16.6
**177**	1.3±0.1	161.7±5.4	1.5±0.2	162.8±7.4	2.1±0.4	169.8±10.6	1.7±0.2	189.3±14.0	1.8±0.2	187.5±15.2
**207**	1.2±0.1	145.8±6.3	1.4±0.1	151.2±2.1	2.7±0.4	147.8±9.9	1.7±0.3	197.2±14.3	1.7±0.1	188.5±19.6
**252**	1.0±0.0	133.7±7.8	1.1±0.1	133.3±9.4	1.6±0.3	123.2±11.9	1.2±0.2	160.0±26.6	1.2±0.2	154.7±13.9
**315**	1.0±0.1	126.7±6.6	1.1±0.1	115.0±9.2	2.6±0.5	112.0±12.8	1.1±0.1	146.0±26.6	1.6±0.3	133.2±16.0

### Electrolytes

**Figure 5:**
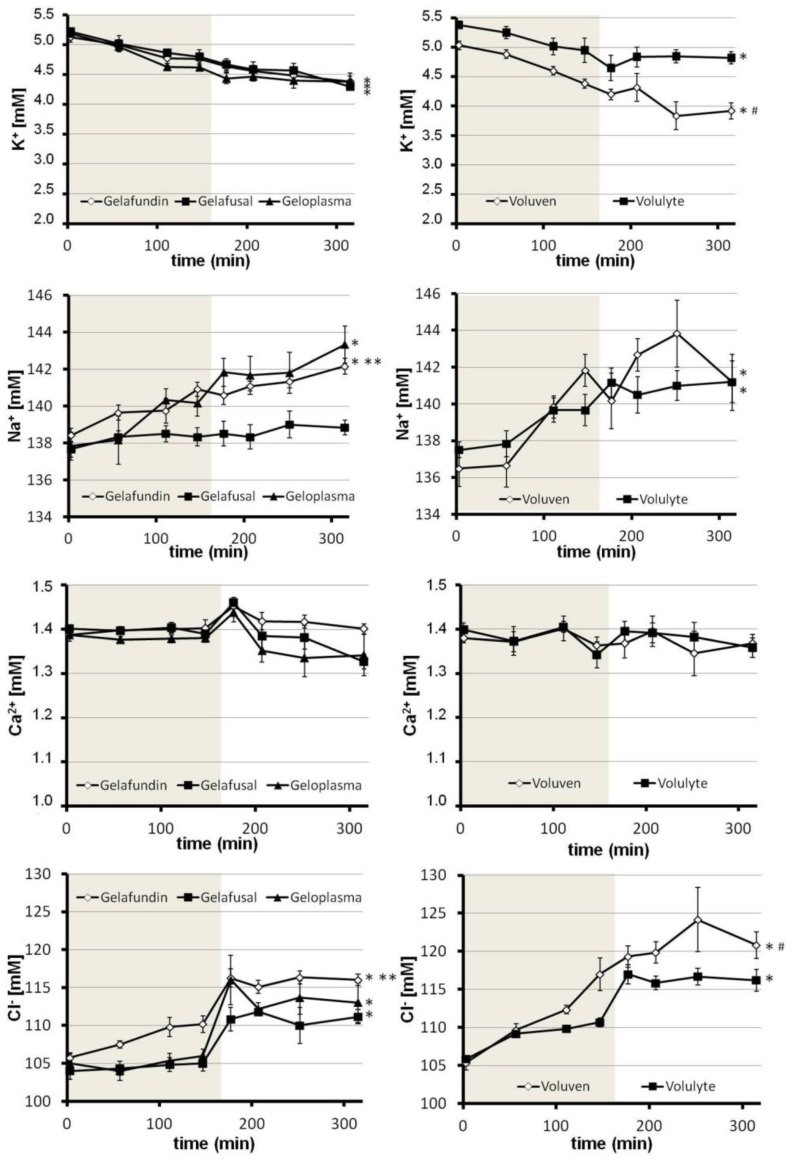
Effects of different volume replacement solutions on electrolyte parameters during and subsequent to normovolemic hemodilution. Rats underwent normovolemic hemodilution to a final hematocrit of 10% for 162 min (phase of hemodilution: light grey) with either Gelafundin, Gelafusal, Geloplasma, Voluven or Volulyte and were subsequently observed for 150 min. Electrolytes were determined in arterial blood samples at the points indicated. * <0.05 compared with the value measured at 3 min, in the respective group. # <0.05 (Voluven vs. Volulyte) ** <0.05 (Gelafundin vs. Gelafusal).

The plasma concentration of K^+^ steadily decreased throughout the experiment in all 5 groups from an initial value of approximately 5.3 mM to 4.8 mM (Volulyte), 4.4 mM (Gelafundin, Geloplasma), 4.3 mM (Gelafusal) and 3.9 mM (Voluven), respectively (Figure 5).

The Na^+^ concentration in the Gelafundin, Geloplasma, Voluven and Volulyte groups steadily rose after beginning of hemodilution from initial values around 138 mM to reach final values of 141 mM (Voluven, Volulyte), 142 mM (Gelafundin) and 143 mM (Geloplasma) at the end of the experiment (Figure 5). The Na^+^ concentration in the Gelafusal group stayed around 138 mM during the whole experimental time.

For the phase of dilution, the plasma Ca^2+^ concentration remained between 1.34 mM and 1.41 mM in all groups studied (Figure 5). After cessation of hemodilution, there was an increase to values around 1.45 mM in those groups diluted with Gelafundin, Gelafusal and Geloplasma, respectively. In the Voluven and Volulyte groups, Ca^2+^ concentration remained constant in this phase.

Starting from an initial value of approximately 105 mM, the plasma Cl^−^ concentration stayed constant (Gelafusal and Geloplasma) or slightly increased (Gelafundin, Voluven and Volulyte) during the phase of dilution (Figure 5). In the subsequent observation time, the values rapidly increased reaching final values of 111 mM (Gelafusal), 113 mM (Geloplasma), 116 mM (Gelafundin and Volulyte) and 120 mM (Voluven), respectively.

### Electrolytes, Glucose and Succinylated Gelatin in Urine and Kidney Injury

Urinary electrolytes, glucose, and succinylated gelatin as well as kidney injury were only studied in the Voluven and Volulyte group as well as in 6 animals of the Gelafundin group.

Animals of the Gelafundin and Volulyte groups excreted an urine volume of approximately 2 ml both during the phase of dilution as well as during the observation time (Table 3). In contrast, in the Voluven group, urine excreted amounted to about double that volume, i.e., 5 ml and 3 ml, respectively.

**Table 3:** Effects of different volume replacement solutions on electrolyte and metabolic parameters as well as pH during and subsequent to normovolemic hemodilution in urine.

Volumereplacementsolution	Interval	Urinevolume [µl]	Parameter								
			pH	cK^+^[mM]	K^+^[µmol]	cNa^+^[mM]	Na^+^[µmol]	cCl^−^[mM]	Cl^−^[µmol]	cGlucose[mg/dl]	Glucose[mg]
**Voluven**	Initial	217±114	7.2±0.2	62.9±3.1	–	54.0±1.7	–	49.0±4.1	–	18.0±2.0	–
	Dilution	5267±666	6.2±0.1	125.3±5.1	612.6±0.0	103.0±2.5	553.4±0.2	182.0±6.4	906.1±0.1	425.0±80.5	9.5±4.0
	Observationtime	3433±593	6.1±0.1	132.2±5.8	458.91±0.1	104.5±5.7	408.1±0.1	205.0±11.2	780.8±0.2	321.5±43.6	9.0±3.7
**Volulyte**	Initial	630±99	7.6±0.1	64.0±5.2	–	71.5±4.8	–	65.0±9.3	–	8.4±0.4	–
	Dilution	2540±244	6.4±0.2	98.4±3.8	265.8±0.0	62.0±2.8	175.9±0.0	90.5±4.9	260.3±0.1	453.0±71.4	12.7±6.8
	Observationtime	2580±480	6.5±0.2	130.8±5.1	353.1±0.1	67.5±3.1	201.2±0.1	136.5±8.8	384.4±0.1	435.6±91.3	14.7±10.2
**Gelafundin**	Initial	600±183	7.2±0.2	92.8±3.4	–	71.5±2.5	–	85.0±5.4	–	15.0±0.8	–
	Dilution	2242±114	6.1±0.1	168.3±4.6	378.9±0.0	50.0±1.3	113.5±0.0	81.5±5.1	183.7±0.0	40.0±1.0	0.9±0.1
	Observationtime	1867±182	6.1±0.1	206.7±3.1	381.9±0.0	64.5±2.2	123.0±0.0	226.0±7.6	412.3±0.0	58.0±4.4	1.0±0.2

Note: Calculations were made based on the values of the six individual animals. Therefore calculations using mean values may differ.

In all groups studied, during the phase of hemodilution, the urinary pH decreased from neutral to acidic values of 6.2 to 6.4, remaining close to these values during the observation time (Table 3).

As compared to the initial values of around 60 to 90 mM, the K^+^ concentration roughly doubled in the urine collected during hemodilution and was only somewhat further increased in the urine collected during the observation time (Table 3). Taken the urine volume of the respective interval into consideration, the total amount of K^+^ excreted was the highest in the Voluven group (about 500 µmol, as compared to Volulyte 265 µmol, Gelafundin 380 µmol); with little differences in the amount excreted between the phase of dilution and the observation time.

There were only little changes in the urinary Na^+^ concentration in the Gelafundin and Volulyte groups (Table 3). Only in the Voluven group, the urinary Na^+^ concentration was increased from an initial value of ca. 50 mM to values of ca. 100 mM, both in the urine collected during the phase of dilution and in the observation time. The absolute amounts of Na^+^ excreted were about 100 µmol for Gelafundin, 200 µmol for Volulyte and 450 µmol for Voluven. As with K^+^, differences between intervals were small.

In urine collected during the phase of dilution, only animals having received Voluven displayed an altered Cl^−^ concentration which was two to three times as high as in the initial sample (50 to 85 mM, Table 3). In the samples collected during the observation time, Cl^−^ concentration stayed approximately the same in the Voluven and Volulyte group and the Gelafundin group’s value doubled. The absolut amount of Cl^−^ excreted in the first interval was 200 µmol (Volulyte and Gelafundin) and 800 µmol (Voluven), respectively, whereas in the last interval, values doubled in the Volulyte and Gelafundin groups but the amount of Cl^−^ excreted stayed nearly the same in the Voluven group.

During the phase of dilution, urinary glucose concentration increased about 40-fold in the Voluven and Volulyte group and 5-fold in the Gelafundin group as compared to the initial concentration of 10 mg/dl (Table 3). In the observation time, these values stayed constant. Considering urine volume, 10 mg glucose were excreted in the Voluven and Volulyte group and 1 mg in the Gelafundin group in each interval.

NMR studies of the Gelafundin animals’ urine showed traces of succinylated gelatin (4.3 mg/ml, absolute amount 3.9 mg) in the initial urine sample (Table 4). During the phase of dilution, animals’ urine contained an average concentration of succinylated gelatin of 134.3 mg/ml, which adds up to an amount of 301.8 mg. The concentration inn urine collected during the time of dilution was 88.6 mg/ml on average which corresponds to an excreted amount of 165.3 mg. Animals having been substituted with Voluven hardly excreted any succinylated gelatin in the observation time (0.9 mg/ml, absolute amount 1.5 mg).

**Table 4:** Quantification of succinylated gelatin in urine samples taken during and subsequent to normovolemic hemodilution.

Sample	Urinevolume [µl]	Concentration ofsuccinylated gelatin [mg/ml]	Quantification ofsuccinylated gelatin [mg]	Percentage ofamount excreted [%]
Initial urine Gelafundin	600±183	4.3±0.7	3.9±0.5	–
Dilution Gelafundin	2242±114	134.3±12.9	301.8±29.6	56
Observation time Gelafundin	1867±182	88.6±5.5	165.3±16.1	31
Observation time Voluven	3433±593	0.9±0.5	1.5±0.1	–

Note: Calculations were made based on the values of the six individual animals. Therefore calculations using mean values may differ.

Calculations concerning the percental excretion were made assuming that the animal had received 540 mg succinylated gelatin during the phase of dilution.

Histological evaluation of the kidneys displayed vacoulization of proximal tubular cells in all groups studied (data not shown). Kidneys of the Gelafundin group tended to be more affected than those of the HES groups (Voluven and Volulyte). No differences were visible between the Voluven and Volulyte group.

## Discussion

The three volume replacement solutions employed in this study on the basis of succinylated gelatin as colloid osmotic substance differ with regard to electrolytes and metabolizable anions (Table 1). Gelafusal as well as Geloplasma contain a metabolizable anion, acetate (27 mM) and lactate (30 mM), respectively, whereas Gelafundin does not. Apart from the presence of an organic anion, Gelafusal includes the electrolytes Na^+^, K^+^, Ca^2+^ and Cl^−^ as does Geloplasma except for Ca^2+^. In contrast, Gelafundin is only composed of Na^+^ and Cl^−^ apart from succinylated gelatin. For those two solutions containing HES as colloid osmotic substance, there are also differences in the composition of the crystalloid carrier solution. Voluven only contains Na^+^ and Cl^−^ as electrolytes and is therefore the counterpart to Gelafundin. Volulyte includes apart from these two ions K^+^, Mg^2+^ as well as acetate (34 mM).

Expectedly, animals treated with solutions containing a metabolizable anion (i.e., Gelafusal, Geloplasma or Volulyte) experienced normalization of pH in terms of a slight alkalization. Unexpectedly, so did animals that had been diluted by using Gelafundin or Voluven, which do not include any metabolizable anion. Despite the fact, that Gelafusal, Geloplasma and Volulyte contain K^+^, in contrast to Gelafundin and Voluven, plasma K^+^ concentration declined continuously in all groups examined. On the other hand, the plasma Cl^−^ concentration increased continuously independent of the solution used for dilution, even though two of the solutions (Gelafusal and Geloplasma) displayed subphysiological Cl^−^ concentrations. Irrespective of the marked differences in their compositions, however, all of the volume replacement solutions tested (Gelafundin, Gelafusal, Geloplasma, Voluven and Volulyte) turned out to be suitable for severe normovolemic hemodilution.

Conditioned by a light initial respiratory depression due to anesthesia with isoflurane [27], [28], a mild respiratory acidosis existed at the beginning of the experiments in all animals studied (Figure 4) which, however, normalized until the end of hemodilution (decrease in pCO_2_ to values around 40 mmHg, Figure 4). In those animals substituted with either Gelafusal, Geloplasma or Volulyte there was not any indication for a metabolic disturbance of the acid-base status during and following hemodilution (base excess around ±2 mM, Figure 4). This behaviour is clearly in line with the concept of metabolizable anions stabilizing plasma pH due to bicarbonate production during their degradation, thereby antagonizing a suspected dilutional acidosis [11]. On the other hand, according to the concept of dilutional acidosis, one should expect a drastic decrease in HCO_3_^−^ and thus a decrease in base excess and pH upon dilution with Gelafundin or Voluven which lack any metabolizable anion [11], [29]. This, however, did not occur (Figure 4). Instead, the base excess and the pH were slightly decreased in those groups but still remained close to the values of the groups mentioned above (Gelafusal, Geloplasma and Volulyte). Only the Voluven group displayed a more distinct decline of the base excess with a value of approximately-7 mM at maximum in the observation time. At the end of the observation time, however, comparable values of pH and base excess were obtained in all groups studied. Thus, independent of the solution used for hemodilution, the major alterations in the acid-base status were due to the resolution of the initial respiratory acidosis. There was only a slight metabolic acidosis occuring especially in the observation phase and which appears to be compensated by a moderate respiratory alkalosis in the end.

A plausible explanation for this unexpected outcome can be derived from alterations in the electrolyte concentrations both in plasma and in urine. Independent of the solutions used for hemodilution and thus independent of the Na^+^, K^+^ and Cl^−^ concentration, plasma Na^+^ and Cl^−^ concentrations increased, plasma K^+^ concentration decreased and the amount of K^+^ excreted into urine increased. These alterations together with the more acidic urinary pH strongly suggest an aldosterone-dependent mechanism, i.e., increased reabsorption of Na^+^ and Cl^−^ and decreased reabsorption of K^+^ and H^+^ in the kidneys. Release of aldosterone can occur within 20 min [30] and may result from intravascular volume depletion, sensed by the renin-angiotensin-system. Loss of intravascular volume is most likely the consequence of the excretion of the respective colloid osmotic substance (succinylated gelatin or HES) into the urine, resulting in a) an enhancement of diuresis and b) a shift of water from the extracellular into the intracellular compartment. For both colloids a rather short half life has been described (in man: succinylated gelatin: approximately 150 min [1], [31]; HES: 180 min [32]). Due to the small average molecular weight of succinylated gelatin of 30 kDa, these molecules can readily pass the renal barrier. The rapid elimination of about 60% of the total amount of succinylated gelatin infused in the phase of dilution and 30% in the subsequent observation time is in line with these behaviour (Table 4). HES has an initial molecular weight of 130 kDa and thus should not pass the renal barrier immediatly. However, rats have a high activity of endogenous α-amylase, thus soon generating fragments of HES molecules [33]. The excretion of either colloid osmotic substance should lead to enhanced diuresis. Furthermore, in both HES groups (Voluven and Volulyte), a relatively large amount of glucose was excreted (10 mg per group and interval, see below), which may further increase diuresis and additionally contributed to an enhanced urine volume. Loss of succinylated gelatin and HES in the urine should result in a reduced colloid osmotic pressure in the vascular system. This should lead to water influx into cells and thus further enhance intravascular volume depletion. The slight decrease in MAP observed in each group independent of the solution employed may be in line with such a mechanism.

While most alterations in the electrolyte concentrations in plasma or in the electrolyte amounts excreted into urine are strongly suggestive for an aldosterone-dependent response, an increased Cl^−^ (all groups) and a constant (Volulyte and Gelafundin) or even increased Na^+^ excretion (Voluven) appears not to be compatible with such a mechanism. However, these variations are presumably attributed to the carrier solution’s composition with supraphysiological concentrations of both Cl^−^ (Voluven 154 mM, Volulyte 110 mM, Gelafundin 120 mM, Table 1) and Na^+^ (Voluven and Gelafundin 154 mM, Volulyte 137 mM, Table 1) and especially to the hypertonicity of Voluven (304 mOsm/kg; for comparison: physiological osmolality in Wistar rats 290 mOsm/kg [34], Volulyte 283 mOsm/kg and Gelafundin 265 mOsm/kg, Table 1). The hypertonicity of Voluven should also account for the increased urine volume in this group (Table 3).

Other mechanisms for normalization of plasma pH are rather unlikely. For instance, one could assume an increased activation of the renal H^+^/K^+^ antiporter due to dilution with a K^+^-free solution (Gelafundin and Voluven) and thus a decreasing plasma K^+^ concentration. However, plasma K^+^ concentration also declined at a comparable rate in all other groups (Gelafusal, Geloplasma and Volulyte), although these solutions contain K^+^ in an almost physiological range. Additionally, due to the postulated enhanced K^+^ reabsorption via the H^+^/K^+^ antiporter, the amount of K^+^ excreted should have been decreased. This was not the case. Instead, the urinary amount of K^+^ increased both during the phase of dilution and during the observation time in all experimental groups studied (Table 3).

In the HES groups, but also in the Gelafundin group, vacuolization of proximal tubular cells was observed, a histopathologically finding generally known as osmotic nephrosis. According to the classification of Janssen et al., this alteration only corresponds to a slight damage [35], whose presence does not necessarily impair renal function [36]. Actually, the observed responses in electrolyte and acid-base homeostasis strongly support this assumption. Osmotic nephrosis due to treatment with HES has already been shown in the past [37]–[39]. The present results suggest that such an injury may also result from application of succinylated gelatin, in line with few existing reports about adverse renal effects elicited by solutions containing gelatin [40].

The elevated excretion of glucose in both HES groups is in line with previous findings obtained in a model of an isolated perfused pig kidney model [37]. Since renal damage is unlikely to account for this observation, competitive inhibition of glucose re-uptake by the HES molecules may be responsible for the impaired renal uptake of glucose. In support of such a mechanism, only minor amounts of glucose were found in the urine of the Gelafundin group.

The slight increase in Ca^2+^ concentration shortly before cessation of dilution and the subsequent decline to the initial level in the groups containing succinylated gelatin cannot be explained by the composition of the respective carrier solution (Table 1). The alterations, however, were only small and no differences were observed between the Gelafusal group, which contains Ca^2+^, and both other groups lacking this cation (Gelafundin and Geloplasma).

As can be seen from the reliable achievement of the critical hematocrit in any group, there were no differences in the quality of dilution between the solutions employed (Figure 2 for those animals having been diluted with succinylated gelatin). In all groups, all animals survived the experiment, although they experienced maximal hemodilution (until the critical hematocrit). We have no explanations for the observed sudden changes in Ca^2+^ concentration, base excess and pCO_2_ after cessation of dilution but they may be related to the achievment of the critical hematocrit. Furthermore, in the groups diluted without exogenous lactate (all except Geloplasma), the plasma lactate concentration remained below 1.9 mM until the end of the experiment (Table 2). This indicates that the animals did not suffer from overt ischemia despite of the severe hemodilution applied. There was no evidence for any impairment due to an increased oxygen demand as suggested for the administration of lactate [41] or for short-term drops in blood pressure due to vasodilatating effects as proposed for acetate [11], [42].

In our study we decided not to use a hemorrhagic shock model, but a model of severe normovolemic hemodilution. Our goal was to study the influence of the crystalloid carrier solution on acid-base status rather than the effect of the colloid used for fluid replacement on hemodynamics. The model of normovolemic hemodilution to a final hematocrit of 10% provides the opportunity of infusing a maximal amount of volume replacement solution possible. In addition, the obtained results are not subjected to acid-base disturbances like metabolic acidosis as would be expected in a hemorrhagic shock model. On the other hand, due to the removal of large amounts of proteins and other osmotically active substances in the course of normovolemic hemodilution another limitation is the need to employ a colloid-based volume replacement solution instead of an only crystalloid-based solution to maintain intravascular oncotic pressure.

The current findings clearly indicate that metabolizable anions such as lactate or acetate as well as Ca^2+^ and K^+^ being present in the carrier solution do not bring further improvement even during severe normovolemic hemodilution. Thus, with regard to the composition of the carrier solution, there seem to be only minimal requirements, which are isoosmolarity on the one hand and the presence of Na^+^ together with Cl^−^ on the other hand. This conclusion, however, is only valid if succinylated gelatin or HES is used as colloid osmotic substance.

## References

[1] Mitra S, Khandelwal P (2009) Are all colloids same? How to select the right colloid? Indian J Anaesth 53: 592–607. 20640110 PMC2900092

[2] Perel P, Roberts I (2012) Colloids versus crystalloids for fluid resuscitation in critically ill patients. Cochrane Database Syst Rev 6: CD000567. 10.1002/14651858.CD000567.pub5 22696320

[3] Choi PT, Yip G, Quinonez LG, Cook DJ (1999) Crystalloids vs. colloids in fluid resuscitation: a systematic review. Crit Care Med 27: 200–210. 9934917 10.1097/00003246-199901000-00053

[4] Levi M, Jonge E (2007) Clinical relevance of the effects of plasma expanders on coagulation. Semin Thromb Hemost 33: 810–815. 18175286 10.1055/s-2007-1000370

[5] Nolan J (1999) Fluid replacement. Br Med Bull 55: 821–843. 10746333 10.1258/0007142991902808

[6] Mizzi A, Tran T, Karlnoski R, Anderson A, Mangar D, et al (2011) Voluven, a new colloid solution. Anesthesiol Clin 29: 547–555. 21871410 10.1016/j.anclin.2011.05.012

[7] Forster H (1997) [Hydroxyethyl starch as a plasma substitute]. Krankenpfl J 35: 497–506. 9470689

[8] Westphal M, James MF, Kozek-Langenecker S, Stocker R, Guidet B, et al (2009) Hydroxyethyl starches: different products–different effects. Anesthesiology 111: 187–202. 19512862 10.1097/ALN.0b013e3181a7ec82

[9] Wiedermann CJ (2004) Hydroxyethyl starch–can the safety problems be ignored? Wien Klin Wochenschr 116: 583–594. 15515874 10.1007/s00508-004-0237-3

[10] Rehm M, Orth V, Scheingraber S, Kreimeier U, Brechtelsbauer H, et al (2000) Acid-base changes caused by 5% albumin versus 6% hydroxyethyl starch solution in patients undergoing acute normovolemic hemodilution: a randomized prospective study. Anesthesiology 93: 1174–1183. 11046202 10.1097/00000542-200011000-00007

[11] Zander R (2009) Fluid Management-Second expanded edition. Melsungen: Bibliomed-Medizinische Verlagsgesellschaft mbH. 15–31 p.

[12] Mortelmans YJ, Vermaut G, Verbruggen AM, Arnout JM, Vermylen J, et al (1995) Effects of 6% hydroxyethyl starch and 3% modified fluid gelatin on intravascular volume and coagulation during intraoperative hemodilution. Anesth Analg 81: 1235–1242. 7486110 10.1097/00000539-199512000-00020

[13] Freyburger G, Dubreuil M, Boisseau MR, Janvier G (1996) Rheological properties of commonly used plasma substitutes during preoperative normovolaemic acute haemodilution. Br J Anaesth 76: 519–525. 8652324 10.1093/bja/76.4.519

[14] Casutt M, Kristoffy A, Schuepfer G, Spahn DR, Konrad C (2010) Effects on coagulation of balanced (130/0.42) and non-balanced (130/0.4) hydroxyethyl starch or gelatin compared with balanced Ringer’s solution: an in vitro study using two different viscoelastic coagulation tests ROTEMTM and SONOCLOTTM. Br J Anaesth 105: 273–281. 20659913 10.1093/bja/aeq173

[15] Godier A, Durand M, Smadja D, Jeandel T, Emmerich J, et al (2010) Maize- or potato-derived hydroxyethyl starches: is there any thromboelastometric difference? Acta Anaesthesiol Scand 54: 1241–1247. 20840513 10.1111/j.1399-6576.2010.02306.x

[16] Lobo DN, Stanga Z, Aloysius MM, Wicks C, Nunes QM, et al (2010) Effect of volume loading with 1 liter intravenous infusions of 0.9% saline, 4% succinylated gelatine (Gelofusine) and 6% hydroxyethyl starch (Voluven) on blood volume and endocrine responses: a randomized, three-way crossover study in healthy volunteers. Crit Care Med 38: 464–470. 19789444 10.1097/CCM.0b013e3181bc80f1

[17] Margarido CB, Margarido NF, Otsuki DA, Fantoni DT, Marumo CK, et al (2007) Pulmonary function is better preserved in pigs when acute normovolemic hemodilution is achieved with hydroxyethyl starch versus lactated Ringer's solution. Shock 27: 390–396. 17414421 10.1097/01.shk.0000245026.01365.55

[18] Haas T, Fries D, Holz C, Innerhofer P, Streif W, et al (2008) Less impairment of hemostasis and reduced blood loss in pigs after resuscitation from hemorrhagic shock using the small-volume concept with hypertonic saline/hydroxyethyl starch as compared to administration of 4% gelatin or 6% hydroxyethyl starch solution. Anesth Analg 106: 1078–1086. 18349176 10.1213/ane.0b013e318165df18

[19] Dubniks M, Persson J, Grande PO (2007) Plasma volume expansion of 5% albumin, 4% gelatin, 6% HES 130/0.4, and normal saline under increased microvascular permeability in the rat. Intensive Care Med 33: 293–299. 17119921 10.1007/s00134-006-0454-5

[20] Base EM, Standl T, Lassnigg A, Skhirtladze K, Jungheinrich C, et al (2011) Efficacy and safety of hydroxyethyl starch 6% 130/0.4 in a balanced electrolyte solution (Volulyte) during cardiac surgery. J Cardiothorac Vasc Anesth 25: 407–414. 21345699 10.1053/j.jvca.2010.12.005

[21] Lehmann L, Bendel S, Uehlinger DE, Takala J, Schafer M, et al (2012) Randomized, double-blind trial of the effect of fluid composition on electrolyte, Acid-base, and fluid homeostasis in patients early after subarachnoid hemorrhage. Neurocrit Care 18: 5–12. 10.1007/s12028-012-9764-3 22872427

[22] Aksu U, Bezemer R, Demirci C, Ince C (2012) Acute effects of balanced versus unbalanced colloid resuscitation on renal macrocirculatory and microcirculatory perfusion during endotoxemic shock. Shock 37: 205–209. 22089195 10.1097/SHK.0b013e31823ca89c

[23] Almac E, Aksu U, Bezemer R, Jong W, Kandil A, et al (2012) The acute effects of acetate-balanced colloid and crystalloid resuscitation on renal oxygenation in a rat model of hemorrhagic shock. Resuscitation 83: 1166–1172. 22353638 10.1016/j.resuscitation.2012.02.011

[24] Council EPaE (2010) European Commission (2010) Directive 2010/63/EU on the protection of animals used for scientific purposes.

[25] Johannes T, Mik EG, Nohe B, Unertl KE, Ince C (2007) Acute decrease in renal microvascular PO2 during acute normovolemic hemodilution. Am J Physiol Renal Physiol 292: F796–803. 17077389 10.1152/ajprenal.00206.2006

[26] Yang ZJ, Price CD, Bosco G, Tucci M, El-Badri NS, et al (2008) The effect of isovolemic hemodilution with oxycyte, a perfluorocarbon emulsion, on cerebral blood flow in rats. PLoS One 3: e2010. 18431491 10.1371/journal.pone.0002010 PMC2291566

[27] Dardai E, Heavner JE (1987) Respiratory and cardiovascular effects of halothane, isoflurane and enflurane delivered via a Jackson-Rees breathing system in temperature controlled and uncontrolled rats. Methods Find Exp Clin Pharmacol 9: 717–720. 3448450

[28] Imai A, Steffey EP, Farver TB, Ilkiw JE (1999) Assessment of isoflurane-induced anesthesia in ferrets and rats. Am J Vet Res 60: 1577–1583. 10622172

[29] Zander R (2006) Infusion fluids: why should they be balanced solutions? EJHP Practice 12: 60–62.

[30] Oberleithner H, Weigt M, Westphale HJ, Wang W (1987) Aldosterone activates Na+/H+ exchange and raises cytoplasmic pH in target cells of the amphibian kidney. Proc Natl Acad Sci U S A 84: 1464–1468. 3029782 10.1073/pnas.84.5.1464 PMC304451

[31] Edwards JD, Nightingale P, Wilkins RG, Faragher EB (1989) Hemodynamic and oxygen transport response to modified fluid gelatin in critically ill patients. Crit Care Med 17: 996–998. 2791585 10.1097/00003246-198910000-00006

[32] Sirtl C, Laubenthal H, Schimetta W (2008) Volumenersatzlösungen. In: Roissant R, Werner C, Zwißler B, editors. Die Anästhesiologie. Heidelberg: Springer. 384–408.

[33] Förster HA F (1998) Grundlagen der Anwendung von Hydroxyethylstärke-Was ist gesichert, was ist Spekulation? J A I 5: 2–11.

[34] Morris M (1982) Neurohypophyseal response to dehydration in the spontaneously hypertensive rat. Hypertension 4: 161–166. 7061123 10.1161/01.hyp.4.1.161

[35] Janssen CW Jr (1968) Osmotic nephrosis. A clinical and experimental investigation. Acta Chir Scand 134: 481–487. 5731288

[36] Dickenmann M, Oettl T, Mihatsch MJ (2008) Osmotic nephrosis: acute kidney injury with accumulation of proximal tubular lysosomes due to administration of exogenous solutes. Am J Kidney Dis 51: 491–503. 18295066 10.1053/j.ajkd.2007.10.044

[37] Hauet T, Faure JP, Baumert H, Bardou A, Gibelin H, et al (1998) Influence of different colloids on hemodynamic and renal functions: comparative study in an isolated perfused pig kidney model. Transplant Proc 30: 2796–2797. 9745568 10.1016/s0041-1345(98)00812-4

[38] Legendre C, Thervet E, Page B, Percheron A, Noel LH, et al (1993) Hydroxyethylstarch and osmotic-nephrosis-like lesions in kidney transplantation. Lancet 342: 248–249. 10.1016/0140-6736(93)92345-t 7686994

[39] Winkelmayer WC, Glynn RJ, Levin R, Avorn J (2003) Hydroxyethyl starch and change in renal function in patients undergoing coronary artery bypass graft surgery. Kidney Int 64: 1046–1049. 12911555 10.1046/j.1523-1755.2003.00186.x

[40] Kief H, Engelbart K, Arnold G, Bahr H (1968) [Vacuolar reabsorption of native and digested gelatin (so-called osmotic nephrosis)]. Virchows Arch B Cell Pathol 1: 240–250. 4971255

[41] Alpert NR, Root WS (1954) Relationship between excess respiratory metabolism and utilization of intravenously infused sodium racemic lactate and sodium L(-) lactate. Am J Physiol 177: 455–462. 13158595 10.1152/ajplegacy.1954.177.3.455

[42] Frohlich ED (1965) Vascular Effects of the Krebs Intermediate Metabolites. Am J Physiol 208: 149–153. 14253139 10.1152/ajplegacy.1965.208.1.149

