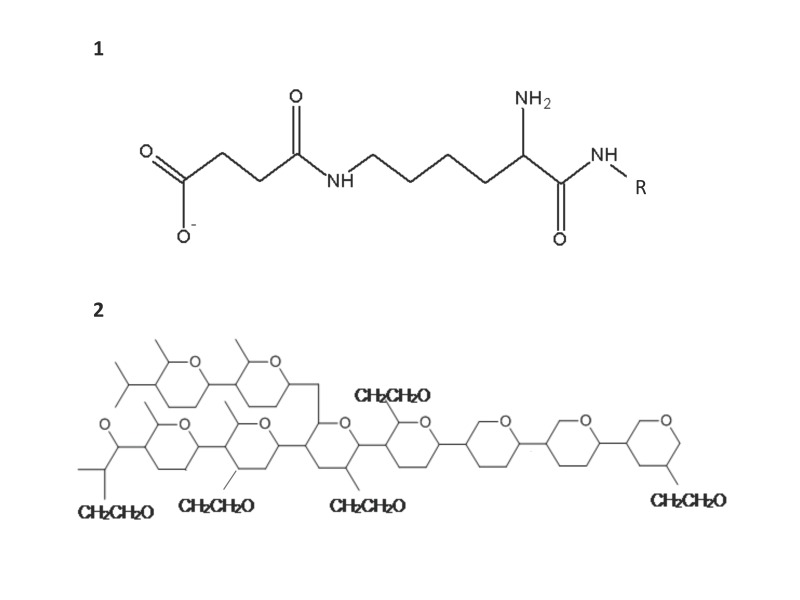# Correction: Acid-Base and Electrolyte Status during Normovolemic Hemodilution with Succinylated Gelatin or HES-Containing Volume Replacement Solutions in Rats

**DOI:** 10.1371/annotation/2881843c-2649-4710-be8f-c7e399cb6660

**Published:** 2013-11-04

**Authors:** Johanna K. Teloh, Katja B. Ferenz, Frank Petrat, Christian Mayer, Herbert de Groot

There were errors in the layout of Table 3, and in Figure 1. The correct versions are available below.

Table 3: 

**Figure:**
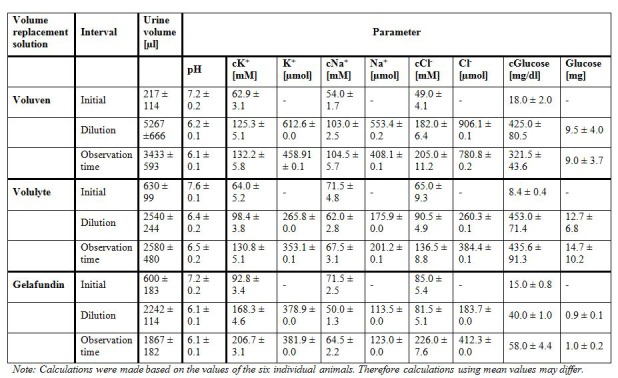


Figure 1: 

**Figure:**